# Inorganic Arsenite Potentiates Vasoconstriction through Calcium Sensitization in Vascular Smooth Muscle

**DOI:** 10.1289/ehp.8000

**Published:** 2005-06-14

**Authors:** Moo-Yeol Lee, Young-Ho Lee, Kyung-Min Lim, Seung-Min Chung, Ok-Nam Bae, Heon Kim, Choong-Ryeol Lee, Jung-Duck Park, Jin-Ho Chung

**Affiliations:** 1 College of Pharmacy, Seoul National University, Seoul, Korea; 2 College of Medicine and BK21 Project for Medical Sciences, Yonsei University, Seoul, Korea; 3 College of Medicine, Chungbuk National University, Cheongju, Korea; 4 Ulsan University Hospital, Ulsan, Korea; 5 College of Medicine, Chung-Ang University, Seoul, Korea

**Keywords:** arsenic, arsenite, blood vessels, calcium sensitization, cardiovascular disease, myosin light chain phosphorylation, vasoconstriction

## Abstract

Chronic exposure to arsenic is well known as the cause of cardiovascular diseases such as hypertension. To investigate the effect of arsenic on blood vessels, we examined whether arsenic affected the contraction of aortic rings in an isolated organ bath system. Treatment with arsenite, a trivalent inorganic species, increased vasoconstriction induced by phenylephrine or serotonin in a concentration-dependent manner. Among the arsenic species tested—arsenite, pentavalent inorganic species (arsenate), monomethylarsonic acid (MMA^V^), and dimethylarsinic acid (DMA^V^)—arsenite was the most potent. Similar effects were also observed in aortic rings without endothelium, suggesting that vascular smooth muscle plays a key role in enhancing vasoconstriction induced by arsenite. This hypercontraction by arsenite was well correlated with the extent of myosin light chain (MLC) phosphorylation stimulated by phenylephrine. Direct Ca^2+^ measurement using fura-2 dye in aortic strips revealed that arsenite enhanced vasoconstriction induced by high K^+^ without concomitant increase in intracellular Ca^2+^ elevation, suggesting that, rather than direct Ca^2+^ elevation, Ca^2+^ sensitization may be a major contributor to the enhanced vasoconstriction by arsenite. Consistent with these *in vitro* results, 2-hr pretreatment of 1.0 mg/kg intravenous arsenite augmented phenylephrine-induced blood pressure increase in conscious rats. All these results suggest that arsenite increases agonist-induced vasoconstriction mediated by MLC phosphorylation in smooth muscles and that calcium sensitization is one of the key mechanisms for the hypercontraction induced by arsenite in blood vessels.

Arsenic is a ubiquitous element distributed in the environment, and millions of people are chronically exposed to arsenic worldwide (Abernathy et al. 1999). Naturally contaminated drinking water is the main source of arsenic exposure, posing potential risk to human health (Nordstrom 2002; Schoen et al. 2004; Smith et al. 2002). Chronic arsenic exposure has been associated with a wide range of illnesses including cancer, hyperkeratosis, diabetes, and cardiovascular disease (Engel et al. 1994; Rossman 2003; Tseng 2004; Yu et al. 1984). Cardiovascular effects of arsenic exposure include hypertension, atherosclerosis, cerebrovascular disease, ischemic heart disease, and peripheral vascular disorders such as black-foot disease (resulting from gangrene caused by obstruction of peripheral blood vessels) in humans (Chen et al. 1995, 1996; Chiou et al. 1997; Rahman et al. 1999; Simeonova et al. 2003; Wang et al. 2002).

Lee et al. (2002) recently suggested that the mechanism for arsenic-induced cardiovascular disease is the increased susceptibility of platelets to aggregate, resulting in enhanced arterial thrombosis. Other mechanisms may also be responsible for the diversity of human cardiovascular disease from chronic arsenic exposure. One possibility is that arsenic may alter the normal vasomotor tone of blood vessels, which rises from contractility of vascular smooth muscle cells.

The contraction of smooth muscle is regulated by mediators such as neural and humoral factors, mechanical forces, and vasoactive substances from endothelial cells. Vascular smooth muscle contraction is triggered primarily by a rise in intracellular free Ca^2+^ concentration (Sanders 2001). Ca^2+^ binds to calmodulin (CaM), allowing Ca^2+^–CaM complex formation, which binds to and activates myosin light chain kinase (MLCK) (Horowitz et al. 1996). The active MLCK catalyzes the phosphorylation of the regulatory myosin light chain (MLC), which then triggers myosin–actin interaction, leading to the shortening of muscle and generation of force. When the intracellular Ca^2+^ concentration returns to a lower level, myosin is dephosphorylated by myosin phosphatase and the muscle relaxes (Somlyo and Somlyo 1994; Walsh et al. 1994).

In addition to the contraction mediated by Ca^2+^-dependent MLCK, it has been recently suggested that smooth muscle contraction is modulated by Ca^2+^ sensitization (Somlyo and Somlyo 2000). This implies that Ca^2+^-dependent contractions occur, but at a lower Ca^2+^ concentration than would be expected for that mediated directly via MLCK (Somlyo and Somlyo 2000). An increase in MLC phosphorylation due to reduced activity of myosin phosphatase appears to result in the hypercontraction of blood vessels, which is a characteristic feature of Ca^2+^ sensitization (Fukata et al. 2001; Uehata et al. 1997). This abnormal hypercontraction is generally known to cause acute vasospasm, micro-circulatory ischemia, increased systemic blood pressure, and ultimately, possible vascular diseases (Bohr et al. 1991; Mohri et al. 1998; Mulvany and Aalkjaer 1990).

Previous epidemiologic studies have reported that peripheral vascular resistance and systemic blood pressure were elevated in populations that had ingested the arsenic-contaminated drinking water (Chen and Yen 1964; Tseng et al. 1995). Carmignani et al. (1985) observed that chronic administration of arsenite to rats and rabbits caused a significant increase in vascular peripheral resistance. These studies suggest the possibility that arsenic may disrupt normal vasomotor function, leading to hypercontraction of blood vessels. Indeed, our previous study demonstrated that arsenic could inhibit acetylcholine-induced vascular relaxation via inhibition of nitric oxide synthase in vascular endothelial cells (Lee et al. 2003). In the process of investigating the effect of arsenic on blood vessels, we observed that arsenic could enhance agonist-induced contraction in an aortic ring organ bath system, suggesting that arsenic could disrupt contractile function in vascular smooth muscles as well. Therefore, in the present study we investigated the mechanism of arsenic-induced vascular dysfunction and its possible contribution to cardiovascular diseases.

## Materials and Methods

### Chemicals.

The following chemicals were purchased from Sigma (St. Louis, MO, USA): sodium arsenite (trivalent inorganic arsenic), sodium arsenate (pentavalent inorganic arsenic), dimethylarsinic acid (DMA^V^), phenylephrine, and serotonin creatinine sulfate. We obtained monomethylarsonic acid (MMA^V^) from Chem Service (West Chester, PA, USA) and anti-MLC and anti-phospho-MLC antibody from Santa Cruz Biotechnology (Santa Cruz, CA, USA). Fura-2/AM was supplied by Molecular Probe (Eugene, OR, USA), and all other reagents used were of the highest purity available.

### Animals.

The entire animal protocol was approved by the Ethics Committee of Animal Service Center at Seoul National University. We used male Sprague-Dawley rats (Dae Han BioLink Co., Seoul, Korea) weighing 300–400 g throughout all experiments. Before the experiments, animals were acclimated for 1 week in the laboratory animal facility maintained at constant temperature and humidity with a 12-hr light/dark cycle. Food and water were provided *ad libitum*.

### Measurement of vasoconstriction in organ bath.

Rats were sacrificed by decapitation and then exsanguinated. We carefully isolated the thoracic aorta and cut it into ring segments. Aortic rings without endothelium were prepared by gently rubbing the intimal surface of the aortic rings with a wooden stick. We then mounted the rings in four-channel organ baths filled with Krebs-Ringer (KR) solution (115.5 mM NaCl, 4.6 mM KCl, 1.2 mM KH_2_PO_4_, 1.2 mM MgSO_4_, 2.5 mM CaCl_2_, 25.0 mM NaHCO_3_, and 11.1 mM glucose, pH 7.4). The organ bath was continuously gassed with 95% O_2_/5% CO_2_ and maintained at 37°C. The rings were stretched gradually to an optimal resting tension of 2 g and equilibrated for 30 min. The change in tension was measured isometrically with Grass FT03 force transducers (Grass Instrument Co., Quincy, MA, USA) and recorded using the AcqKnowledge III computer program (BIOPAC Systems Inc., Goleta, CA, USA).

To investigate the effect of arsenic on vasoconstriction, we treated the aortic rings with arsenic or the vehicle (saline) in minimum essential media (MEM) with 100 U/mL penicillin and 100 μg/mL streptomycin in a 95% air/5% CO_2_ incubator for 14 hr at 37°C. After mounting the aortic rings pretreated with arsenic in organ baths, we induced the contraction by cumulatively adding phenylephrine or serotonin to obtain concentration-contraction curves. In the experiments of high K^+^-induced contraction, 100 mM KCl-containing KR solution prepared by substituting K^+^ with Na^+^ was cumulatively added to the bath to obtain the indicated final concentrations.

### Measurement of MLC phosphorylation.

We measured the extent of MLC phosphorylation using polyacrylamide gel electrophoresis (PAGE) and immunoblot analysis using an anti-phospho-MLC antibody, as previously described (Sakurada et al. 1998). After the aortic rings were pretreated with various concentrations of arsenite for 14 hr, 10^−8^ M phenylephrine was added to the organ bath for 2 min, and then ice-cold acetone with 10% trichloroacetic acid and 10 mM dithiothreitol was immediately added to stop the reaction. The aortic rings were washed with the acetone solution three times, lyophilized overnight, and stored at −70°C until protein extraction. After the dried tissues were cut into small pieces, we extracted proteins in a 50 μL sample buffer containing 8 M urea, 2% sodium dodecyl sulfate, 5% β-mercaptoethanol, 0.01% bromophenol blue, and 62.5 mM Tris-HCl by vortexing for 3 hr at room temperature. Protein extracts were electrophoresed in a 15% polyacrylamide mini-slab gel (Bio-Rad, Hercules, CA, USA) and then transferred onto nitrocellulose membranes in Tris/glycine buffer (25 mM Tris, 192 mM glycine). We detected the MLC level and the extent of phosphorylation with immunoblotting using anti-MLC antibody and anti-phospho-MLC antibody (Santa Cruz Biotechnology), respectively. Immunoreactive bands were visualized by horseradish peroxidase-conjugated secondary antibody (Santa Cruz Biotechnology) and an enhanced chemiluminescence kit (ECL; Amersham, Buckinghamshire, UK). For densitometric analysis of MLC-P/MLC, we determined densities of the corresponding bands using TINA software (Raytest, Straubenhardt, Germany).

### Measurement of intracellular calcium level.

To measure intracellular Ca^2+^ levels, we cut the thoracic aorta without endothelium into spiral strips approximately 8 mm in length and 1 mm in width under a dissecting microscope. After the aortic strips were treated with arsenite for 14 hr, they were exposed to 10 μM acetoxymethyl ester of fura-2 (fura-2/AM) and 0.1% cremophor EL in a Krebs-Henseleit solution [(KH) solution: 119 mM NaCl, 4.6 mM KCl, 1.2 mM KH_2_PO_4_, 1.5 mM MgSO_4_, 2.5 mM CaCl_2_, 25.0 mM NaHCO_3_, and 11 mM glucose, pH 7.4] for 4 hr at room temperature.

We measured the intracellular free Ca^2+^ level based on the method described by Ozaki et al. (1991). Fure-2-loaded muscle strips were held horizontally in the organ chamber of a fluorimeter (CAF-110; Jasco, Tokyo, Japan) filled with KH solution. One end of the muscle strip was connected to a force-displacement transducer to monitor vessel tones. The KH solution was maintained at 37°C and continuously aerated with 95% O_2_/5% CO_2_. Passive tension of 2 g was applied and allowed to equilibrate before measurement. We elicited muscle contractions by changing the media with the KH solution containing 12.5, 25, and 90 mM KCl. Muscle strips were illuminated alternatively at 48 Hz in excitation wavelengths of 340 and 380 nm. We measured the intensity of 500 nm fluorescence emitted by 340 nm excitation (F_340_) and that emitted by 380 nm (F_380_) successively. We calculated the ratio of F_340_ to F_380_ [R(F_340_/F_380_)] as an indicator of intracellular Ca^2+^.

### Measurement of blood pressure change induced by phenylephrine.

Rats were anesthetized with phenobarbital (50 mg/kg, intraperitoneal). We placed a catheter of polyethylene PE-50 tubing (Clay Adams, Sparks, MD, USA) filled with heparinized saline (100 U/mL) in the carotid artery to measure blood pressure, and we placed a catheter of polyethylene PE-10 fused to PE-50 tubing in the jugular vein to administer drugs. Catheters were tunneled subcutaneously and exteriorized at the back of the neck. Wounds were sutured and cleaned with alcohol. Experiments were performed after a 1-day recovery period. On the day of the experiment, the arterial catheter was connected to a pressure transducer (BIOPAC Systems Inc.) and blood pressure was measured using the AcqKnowledge III computer program. We allowed blood pressure to stabilize for a minimum of 30 min before beginning treatment. To determine the effects of arsenite on blood pressure increase induced by phenylephrine, we administered arsenite solution (1.0 mg/kg) by an intravenous bolus injection into the jugular vein. In the controls, equivalent amounts of saline were injected. After 2 hr, we infused the rats with 2.5 μg/kg/min phenylephrine for 3 min via the jugular vein and monitored the change of blood pressure in response to phenylephrine simultaneously. Infusions were performed with a Harvard syringe pump (South Natick, MA, USA) at a rate of 0.1 mL/min.

### Statistical analysis.

We calculated the means and standard errors for all treatment groups. The data were subjected to one- or two-way analysis of variance (ANOVA) followed by Duncan’s multiple range test to determine which means were significantly different from the control. Statistical analysis was performed using SPSS software (SPSS Inc., Chicago, Il, USA). In all cases, we used a *p*-value of < 0.05 to determine significance.

## Results

**Figure 1:**
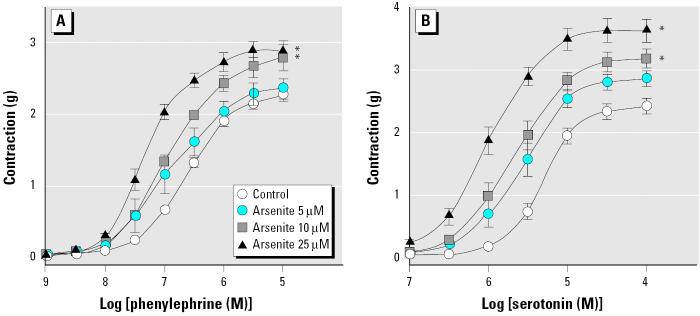
Enhancement of phenylephrine- and serotonin-induced vasoconstriction in aortic rings by arsenite. (*A*) Phenylephrine. (*B*) Serotonin. See “Materials and Methods” for details. Values shown are mean ± SE of four independent experiments. *Significantly different from the corresponding control (*p* < 0.05).

**Figure 2:**
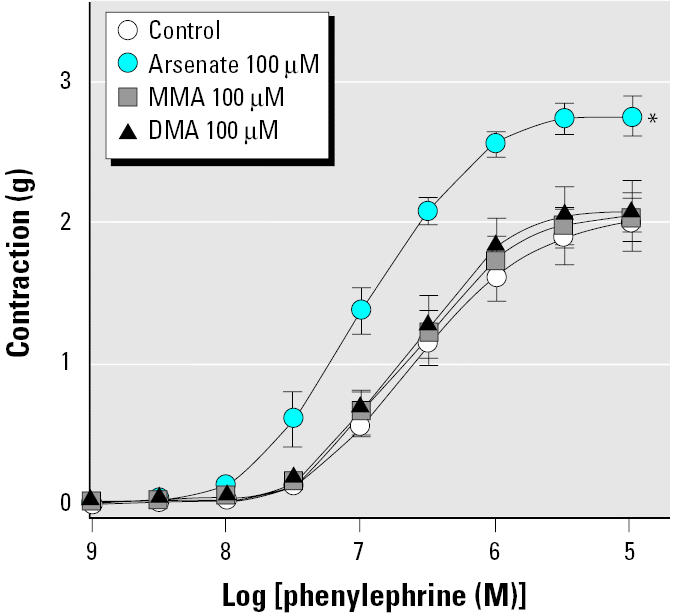
Effect of arsenic species on contraction of aortic rings induced by phenylephrine. See “Materials and Methods” for details. Values shown are mean ± SE of five independent experiments. *Significantly different from the control (*p* < 0.05).

To investigate whether arsenic affects contraction of blood vessels, we treated intact aortic rings with various concentrations of arsenite (trivalent inorganic arsenic) for 14 hr and then added phenylephrine and serotonin cumulatively to obtain concentration-contraction curves. Arsenite alone did not cause any changes in basal vascular tone (data not shown). Arsenite treatment, however, enhanced the contraction induced by both phenylephrine and serotonin in a concentration-dependent manner (Figure 1). We investigated the effects of pentavalent inorganic species (arsenate) and two major metabolites, MMA^V^ and DMA^V^, on phenylephrine-induced constriction (Figure 2). Arsenate did enhance phenylephrine-induced vasoconstriction, but the concentration required was higher than that of arsenite. MMA^V^ and DMA^V^ up to 100 μM failed to affect the phenylephrine-induced vasoconstriction. These results suggest that agonist-induced contraction in blood vessels can be enhanced by arsenic and that arsenite is the most potent form tested.

**Figure 3:**
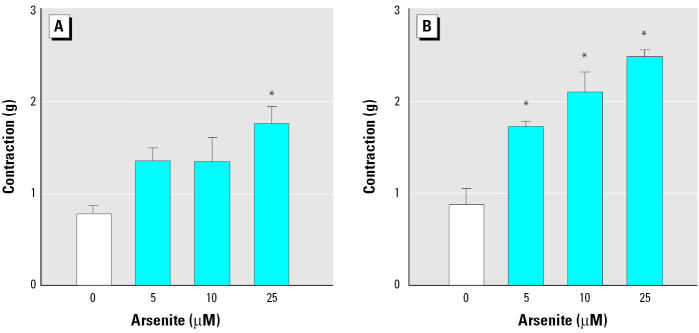
Enhancement of phenylephrine- and serotonin-induced contraction by arsenite in aortic rings without endothelium. (*A*) Phenylephrine (3 x 10^−8^ M). (*B*) Serotonin (10^−6^ M). See “Materials and Methods” for details. Values shown are mean ± SE of four independent experiments. *Significantly different from the control (*p* < 0.05).

**Figure 4:**
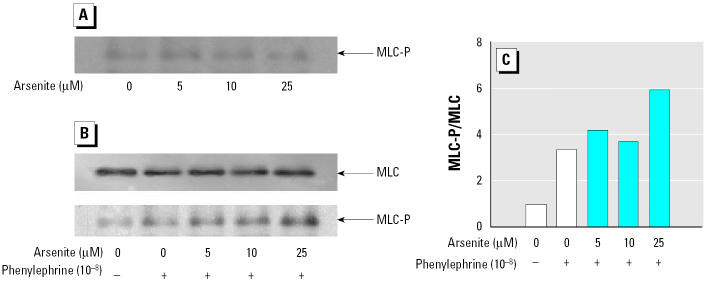
Effect of arsenite on MLC phosphorylation in aortic rings. (*A*) Basal levels of MLC-P. (*B*) MLC and MLC-P levels stimulated by 10^−8^ M phenylephrine. See “Materials and Methods” for details. (*C*) Densitometric analysis of MLC-P/MLC (typical results of one of three independent experiments).

To investigate whether the enhanced contraction induced by arsenite was an endothelium-dependent effect, we performed experiments using aortic rings without endothelium. Treatment with arsenite to aortic rings without endothelium still resulted in a concentration-dependent increment of agonist-induced contraction in blood vessels (Figure 3), suggesting that the enhanced contraction by arsenite is primarily due to hypercontraction of smooth muscles. To examine whether hypercontraction by arsenite is mediated by the phosphorylation of MLC in smooth muscles, we evaluated the effect of arsenite on phenylephrine-induced MLC phosphorylation in aortic rings without endothelium. Arsenite treatment alone did not alter the basal levels of MLC phosphorylation (Figure 4A). Addition of phenylephrine alone did not affect the total MLC levels, but it increased MLC phosphorylation significantly (Figure 4B,C). However, when the aortic rings without endothelium were stimulated with phenylephrine, arsenite treatment resulted in a significant increase in MLC phosphorylation without a concomitant change in MLC levels (Figure 4B,C). These results indicate that arsenite enhances agonist-induced vasoconstriction through MLC phosphorylation in smooth muscles.

**Figure 5:**
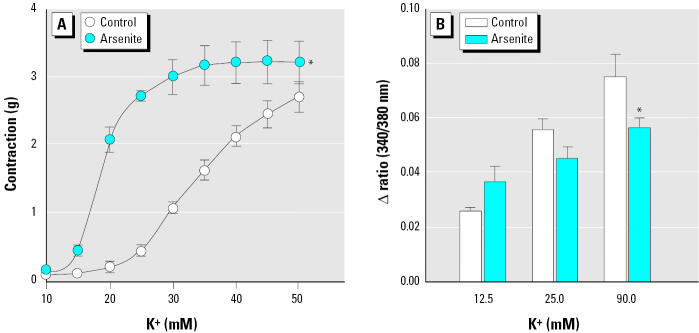
Effect of arsenite on vasoconstriction and intracellular Ca^2+^ levels in the presence of high K^+^. (*A*) Contraction induced by various concentrations of K^+^ in Ca^2+^-free KR solution in aortic rings without endothelium after treatment with arsenite. (*B*) Intracellular Ca^2+^ levels determined in the presence of K^+^ in fura-2 loaded aortic strips without endothelium after treatment with arsenite; values shown are mean ± SE of more than three independent experiments. See “Materials and Methods” for details. *Significantly different from the control group (*p* < 0.05).

To examine whether the increased MLC phosphorylation was mediated by intracellular Ca^2+^ elevation in vascular smooth muscles, we investigated the effects of arsenic on intracellular Ca^2+^ levels when vasoconstriction was initiated by high K^+^ concentration. We used a high concentration of K^+^ to induce contraction in blood vessels, since K^+^ directly induces rapid influx of extracellular Ca^2+^ through voltage-gated Ca^2+^ channels in plasma membrane without involving other signal transduction pathway and thus could serve as a simple alternative tool to investigate the current premise. As shown in Figure 5A, 25 μM arsenite enhanced vasoconstriction induced by 10–50 mM K^+^ in aortic rings without endothelium, which is consistent with the results shown in Figure 3. The next experiment was performed using aortic strips loaded with fura-2 fluorescent dye to investigate whether arsenic increased intracellular Ca^2+^ in the presence of high K^+^. Arsenite, however, did not induce intracellular Ca^2+^ elevation, but rather decreased Ca^2+^ concentration significantly in the presence of 90 mM K^+^ (Figure 5B). These results show that arsenite enhances vasoconstriction without concomitant increase of intracellular Ca^2+^ levels, suggesting that arsenite might increase the contractility of blood vessels via Ca^2+^ sensitization.

**Figure 6:**
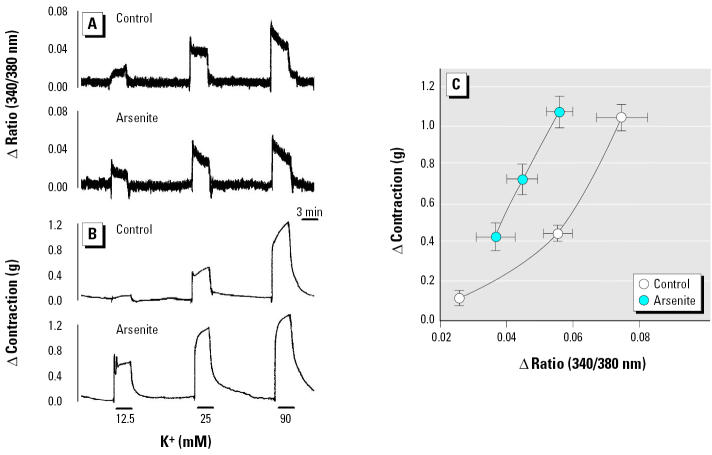
Simultaneous measurement of Ca^2+^ increase and contraction by KCl in aortic strips without endothelium after treatment with arsenite. Representative tracings of intracellular Ca^2+^ increase (*A*) and contraction (*B*) induced by KCl. See “Materials and Methods” for details. (*C*) Ca^2+^ elevation versus the magnitude of contraction; values shown are mean ± SE of seven independent experiments.

To investigate this assumption, we measured smooth muscle contraction and the change of intracellular Ca^2+^ levels simultaneously using fura-2–loaded aortic strips. After the muscle strips were treated with 25 μM arsenite, contraction was induced successively by 12.5, 25, and 90 mM K^+^. Figure 6A and 6B shows a scanned reduction of the tracings from aortic strips. Compared with the control group, arsenite-treated aortic strips showed enhanced contraction without any concomitant increase in intracellular Ca^2+^ elevation (Figure 6A,B). Plotting this result reveals that the aortic strips treated with arsenite showed a steeper slope in the intracellular Ca^2+^ elevation versus contraction relationship (Figure 6C). These results suggest that arsenite might potentiate vasoconstriction by enhancing the Ca^2+^ sensitivity of contractile machinery in smooth muscle.

**Figure 7:**
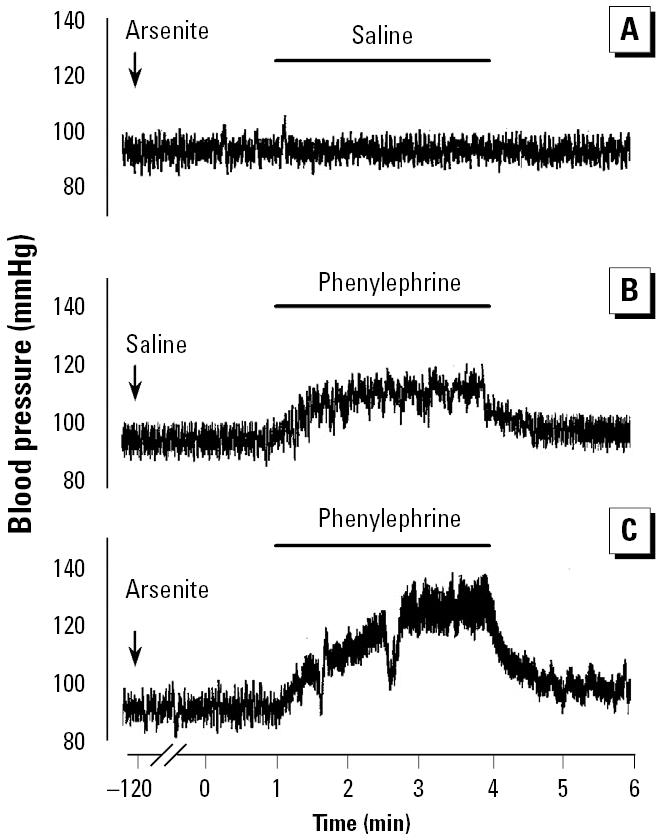
Effect of intravenously administered arsenite on phenylephrine-induced blood pressure increase in rats. (*A*) Saline 2 hr after arsenite exposure. (*B*) Phenylephrine 2 hr after saline exposure (ΔmmHg = 23.0 ± 3.2; mean ± SE of four animals). (*C*) Phenylephrine 2 hr after arsenite exposure (ΔmmHg = 36.8 ± 3.6; mean ± SE of four animals). See “Materials and Methods” for details. Periods of infusion are indicated in each panel. Data are representative tracings of four independent experiments.

To verify the effects of arsenite on blood vessels *in vivo*, we monitored changes in blood pressure after intravenous infusion of phenylephrine into conscious rats (Figure 7). An intravenous bolus of arsenite had no effect on basal blood pressure. When rats were treated with arsenite 2 hr before phenylephrine infusion, the hypertensive effect of phenylephrine was significantly potentiated (23.0 ± 3.2 vs. 36.8 ± 3.6 mmHg, *p* = 0.029; Figure 7B,C). These results suggest that arsenite could induce the enhancement of agonist-induced vasoconstriction *in vivo* and this confirms the previous *in vitro* results (Figure 1)*.*

## Discussion

In the present study we demonstrate the ability of arsenic to enhance contraction of isolated aortic rings from rats. We have shown that arsenite enhances the vascular contraction induced by phenylephrine, serotonin, and high K^+^ in a concentration-dependent manner and that the Ca^2+^ sensitization in smooth muscle largely contributes to arsenite-induced hypercontractility. These *in vitro* results were consistent with *in vivo* results in which arsenite potentiated the hypertensive effect of phenylephrine. Recently, the effect of arsenic on platelets has been suggested as a key mechanism in the development of cardiovascular diseases (Lee et al. 2002). Blood vessels, however, are another important tissue in cardiovascular system. Hyper-contractility of blood vessels disrupts vasomotor tone regulation and makes vasoconstriction predominate, which can induce hypertension, complicating cardiovascular disease. Elevated peripheral resistance has been reported in populations exposed to arsenic-contaminated drinking water and in animals treated with arsenite (Carmignani et al. 1985; Chen and Yen 1964). Our data provide evidence that arsenite could enhance vascular smooth muscle contractility, suggesting that arsenic-induced hypercontraction of blood vessels might be another mechanism for arsenic-associated cardiovascular disease observed in human populations.

Suppression of nitric oxide production in endothelium results in the loss of vasodilator activity, causing vasoconstriction (Moncada et al. 1991), and we previously demonstrated that arsenite disrupts endothelium-dependent vasorelaxation via inhibition of endothelial nitric oxide production (Lee et al. 2002). Pi et al. (2003) also reported that vasoconstriction was increased in aortic rings isolated from arsenate-exposed rabbits. They explained the phenomena as a result of impaired nitric oxide formation. To examine whether the hypercontraction of blood vessels observed (Figure 1) was also mediated by the inhibition of endothelium-derived vasorelaxation activity, we performed experiments in aortic rings without endothelium. Surprisingly, contraction by phenylephrine or serotonin was still enhanced by arsenite treatment (Figure 3), suggesting that the hypercontraction was dependent on smooth muscles in blood vessels. Because arsenite not only interferes with endothelium-dependent vasorelaxation but also enhances smooth muscle-dependent contraction, arsenite treatment could result in overall hypercontraction of blood vessels, leading to a possible increased risk for development of vascular diseases such as hypertension and atherosclerosis (Bohr et al. 1991; Mulvany and Aalkjaer 1990; Rubanyi 1993; Vanhoutte 1997).

As shown in Figure 2, treatment with arsenate (pentavalent inorganic arsenic) also potentiated phenylephrine-induced contraction, whereas MMA^V^ and DMA^V^ showed no significant effect. Arsenate is generally known to exert its toxic effects by replacing phosphate in various biochemical reactions because it has a similar structure and properties to phosphate (Oremland and Stolz 2003). However, it is not certain that such properties are engaged in the enhancement of vasoconstriction shown in this study. Indeed, arsenate can be reduced to arsenite at a comparable rate in cell systems, and the biological effect of arsenate may in part result from its reduction to arsenite (Huang and Lee 1996). Therefore, considering that the effective concentration of arsenate is higher than that of arsenite, it is possible that the effect of arsenate arises from arsenite formed by reduction of arsenate.

Phenylephrine and serotonin act on the α_1_-adrenoceptor and the 5-HT_2_ serotonin receptor, respectively, on aortic smooth muscle cells and thus result in increasing intracellular Ca^2+^ (Bruckner et al. 1984; Peroutka 1984). In contrast to these receptor agonists, a high concentration of K^+^ bypasses receptor-signaling pathways and leads directly to intracellular Ca^2+^ elevation by opening Ca^2+^ channels following membrane depolarization (Chiu et al. 1987). These intracellular Ca^2+^ increases by phenylephrine, serotonin, and high K^+^ result in phosphorylation of MLC leading to vascular smooth muscle contraction. It is widely accepted that the degree of MLC phosphorylation is the essential factor that determines the extent to which smooth muscle contracts (Fukata et al. 2001; Walsh 1994). As shown in Figure 4B and 4C, MLC phosphorylation to phenylephrine was augmented by arsenite, from which it can be concluded that hypercontraction by arsenite is an MLC phosphorylation-dependent effect. In contrast, arsenite alone did not induce the MLC phosphorylation significantly (Figure 4A), consistent with the fact that basal tones of blood vessels were not changed by arsenite treatment alone.

In addition to intracellular Ca^2+^ elevation, the level of responsiveness of contractile machinery to intracellular Ca^2+^ plays a key role in regulating the contractility of smooth muscle cells (Somlyo and Somlyo 2000). Simultaneous measurement of contraction and intracellular Ca^2+^ increase in aortic strips revealed that arsenite potentiated the magnitude of contraction without concomitant increase in Ca^2+^ elevation (Figure 6), suggest- significant contribution of Ca^2+^ sensitization to the hypercontraction induced by arsenite. Previous studies suggested that the mechanism for Ca^2+^ sensitization was mainly due to modulation of MLC phosphatase activity (Fukata et al. 2001; Uehata et al. 1997). That is, if MLC phosphatase was inhibited, the enhanced MLC phosphorylation could be elicited at the same Ca^2+^ concentration, which resulted in hypercontraction. However, it is currently uncertain whether arsenite could inhibit MLC phosphatase directly.

**Figure 8:**
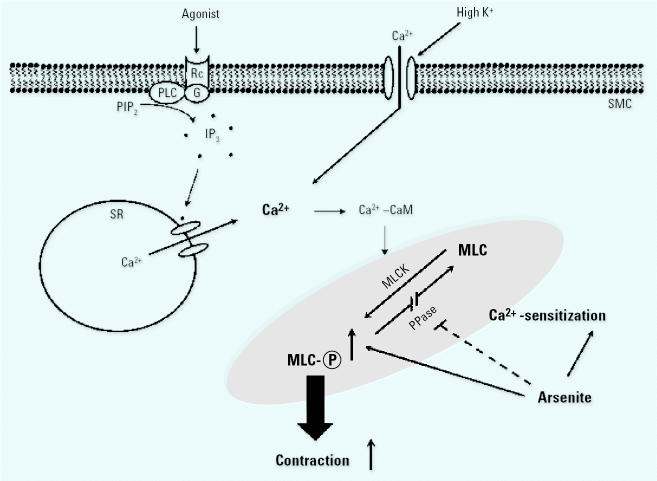
Proposed mechanism for arsenite-induced vasoconstriction. Abbreviations: Ca^2+^–CaM, calcium calmodulin; IP_3_, inositol 1,4,5-trisphosphate; PIP_2_, phosphatidylinositol-4,5-bisphophate; PLC, phospholipase C; PPase, phosphatase; SMC, smooth muscle cells; SR, sarcoplasmic reticulum. Arsenite enhances the contraction of SMC by agonists such as phenylephrine or serotonin mediated through MLC phosphorylation. Arsenite also enhances vasoconstriction induced by high K^+^. The mechanism for this effect is due not to alteration of intracellular Ca^2+^ levels, but to Ca^2+^-sensitization (shaded area) possibly via inhibition of PPase.

In this study, we showed that arsenite causes enhanced vasoconstriction *in vitro* and demonstrated that Ca^2+^ sensitization in smooth muscle is responsible for MLC phosphorylation-dependent hypercontraction arsenite (Figure 8). In our *in vivo* study, arsenite treatment potentiated the hypertensive effect of phenylephrine in rats. These results confirm our *in vitro* observations and suggest that increased vasocontraction may be a contributing factor in the development of cardiovascular diseases in populations exposed to arsenic.
